# Prediction of doxorubicin cardiotoxicity by early detection of subclinical right ventricular dysfunction

**DOI:** 10.1186/s40959-020-00066-8

**Published:** 2020-07-23

**Authors:** Maria Isabel Camara Planek, Ahmad Manshad, Kyaw Hein, Mohamad Hemu, Fatima Ballout, Rajiv Varandani, Parameswaran Venugopal, Tochukwu Okwuosa

**Affiliations:** 1 Department of Medicine, Rush University Medical Center, Chicago, IL 60612 USA; 2 Division of Cardiology, Loyola University Medical Center, Maywood, IL 60153 USA; 3 Division of Nephrology, Rush University Medical Center, Chicago, IL 60612 USA; 4 Chicago College of Osteopathic Medicine at Midwestern University, Downers Grove, IL 60515 USA; 5 Division of Hematology/Oncology, Rush University Medical Center, Chicago, IL 60612 USA; 6 Division of Cardiology, Rush University Medical Center, Chicago, IL 60612 USA

**Keywords:** RV strain, Doxorubicin, Cardiotoxicity

## Abstract

**Background:**

Doxorubicin remains one of the most common causes of cardiotoxicity in patients with lymphoma, leading to significant morbidity and mortality. Early decline in left ventricular (LV) ejection fraction predicts chemotherapy-induced cardiotoxicity and mortality, but limited data exist on doxorubicin-induced subclinical right ventricular (RV) dysfunction. We investigated dose-dependent subclinical doxorubicin-induced RV dysfunction in lymphoma patients.

**Methods:**

Thirty-five patients with adult lymphoma treated with doxorubicin were studied. All patients had normal baseline LV ejection fraction (LVEF > 55%), and no known cardiopulmonary disease. We studied the dose-dependent effect of doxorubicin on RV strain by 2D speckle-tracking echocardiography (STE) using a vendor-independent software (TomTec). Images were analyzed offline by two independent observers blinded to the clinical characteristics of the study population. Baseline LVEF, RV fractional area change (RV FAC), RV free wall strain (RV FWS), and RV global longitudinal strain (RV GLS) were measured prior to chemotherapy initiation and compared with echo studies obtained at a 6-month follow-up interval. Patients served as their own controls. Comparisons between pre- and post-therapy were achieved using paired Student’s t-tests or Chi-Square test.

**Results:**

The Interobserver Intraclass Correlation Coefficient for RV GLS, RV FAC and RV FWS, was 0.87, 0.81 and 0.79, respectively. The mean age was 51 ± 13 years, 40% women, 60% white. The mean cumulative doxorubicin dose was 239 ± 104 mg m^− 2^. There was there was significant decline in RV FAC (47.3 ± 4.4% vs. 43.7 ± 3.9%), RV FWS (− 24.9 ± 3.3 vs. -22.2 ± 2.9), and RV GLS (− 22.4 ± 4.1 vs. -20.6 ± 3.4) (all *p* < 0.01); but no significant decline in LVEF during the 6-month follow up (63.3 ± 6.2% vs. 61.6 ± 11.1%, *p* = 0.374). At cumulative doxorubicin dose ≥200 mg m^− 2^ we found a significant decline in RV FAC (47.0 ± 4.7% vs. 42.2 ± 3.1%, *p* < 0.01), RV FWS (− 24.6 ± 3.6 vs. -21.5 ± 2.4, p < 0.01), and RV GLS (− 22.3 ± 4.5 vs. -20.1 ± 2.9, *p* = 0.03).

**Conclusion:**

In this cohort of adult lymphoma patients, doxorubicin-based therapy was associated with subclinical RV dysfunction, but not LV dysfunction, at a cumulative dose ≥200 mg m^− 2^. Additional studies evaluating the long-term prognostic implications of RV dysfunction in this population are essential.

## Introduction

Anthracyclines are commonly used anti-neoplastic agents in the treatment of a variety of malignancies, including lymphoma; and cardiotoxicity remains an irreversible complication of anthracycline-based chemotherapy. Most current guidelines and clinical trials describe cardiotoxicity as changes in resting cardiac systolic function [1] defined by the current European Society of Medical Oncology [2] consensus statement as left ventricular ejection fraction (LVEF) drop by > 10–15% or to < 50% of total LVEF. The incidence of cardiotoxicity during doxorubicin-based chemotherapy is dose-dependent with higher cumulative doses being associated with higher risk of cardiotoxicity [2], and is yet unpredictable.

In patients receiving doxorubicin, left ventricular myocardial strain assessed by speckle tracking echocardiography (STE) and cardiovascular biomarkers such as natriuretic peptides and high-sensitive cardiac troponins can detect subclinical cardiotoxicity and predict future risk of heart failure [3]. Furthermore, studies have shown that early administration of angiotensin-converting enzyme (ACE)-inhibitors and beta blockers reduce LV dysfunction and cardiac events including sudden death, any cardiac death, and symptomatic heart failure in patients that received high dose doxorubicin [4, 5]. Thus, subclinical/early detection of cardiotoxicity may provide an opportunity for early intervention to prevent progression to advanced heart disease and reduce cardiac mortality.

While the clinical implications of doxorubicin on sub/clinical LV dysfunction have been well established, data are sparse regarding its effects on right ventricular (RV) function. RV function has independent prognostic implications in patients with or without LV systolic dysfunction, and may predict subclinical LV dysfunction in animal models receiving anthracyclines [6].

In this study, we explore overall as well as dose-dependent associations between doxorubicin and subclinical RV function parameters in lymphoma patients using 2D STE.

## Methods

### Study design and population

We included adult patients with a clinical diagnosis of lymphoma who received doxorubicin-based chemotherapy in an urban academic center. The patients were regarded as eligible for this study if they had not received prior anthracycline or radiation therapy to the mediastinum. Patients with previous cardiac diseases including congestive heart failure (CHF), were excluded. The study was reviewed and approved by the Institutional Review Board at Rush University Medical Center.

All patients had normal baseline biplane LVEF > 55%, and each patient served as their own control. Baseline LVEF, RV fractional area change (RV FAC), RV free wall strain (RV FWS), and RV global longitudinal strain (RV GLS) were measured prior to chemotherapy initiation and compared with the echo studies post treatment. The average follow-up period from pre- to post-doxorubicin echocardiography was 6 months.

### Chemotherapeutic regimen

A standard chemotherapeutic regimen of rituximab plus cyclophosphamide, doxorubicin, vincristine, and prednisone (R-CHOP) was administered to each of the patients. All patient receiving R-CHOP received 6 cycles and each cycle was made up of: cyclophosphamide 750 mg/m2; vincristine 1.4 mg/m2; doxorubicin 50 mg/m2 on day 1; prednisone 100 mg on days 1 to 5; and rituximab 375 mg/m2 every 21 days).

### Conventional echocardiography

Comprehensive echocardiographic examinations were carried out and analyzed using General Electric, Vivid 7 Dimension imaging system device (GE Vingmed Ultrasound AS, Horten, Norway) with a 3.5 MHz transducer in accordance with the standard recommendations of the American Society of Echocardiography guidelines [7]. LVEF was measured by biplane Simpsons method in apical 4- and 2-chamber views with 3 consecutive heart cycles recorded for each view.

### Speckle tracking echocardiography

**Fig. 1:**
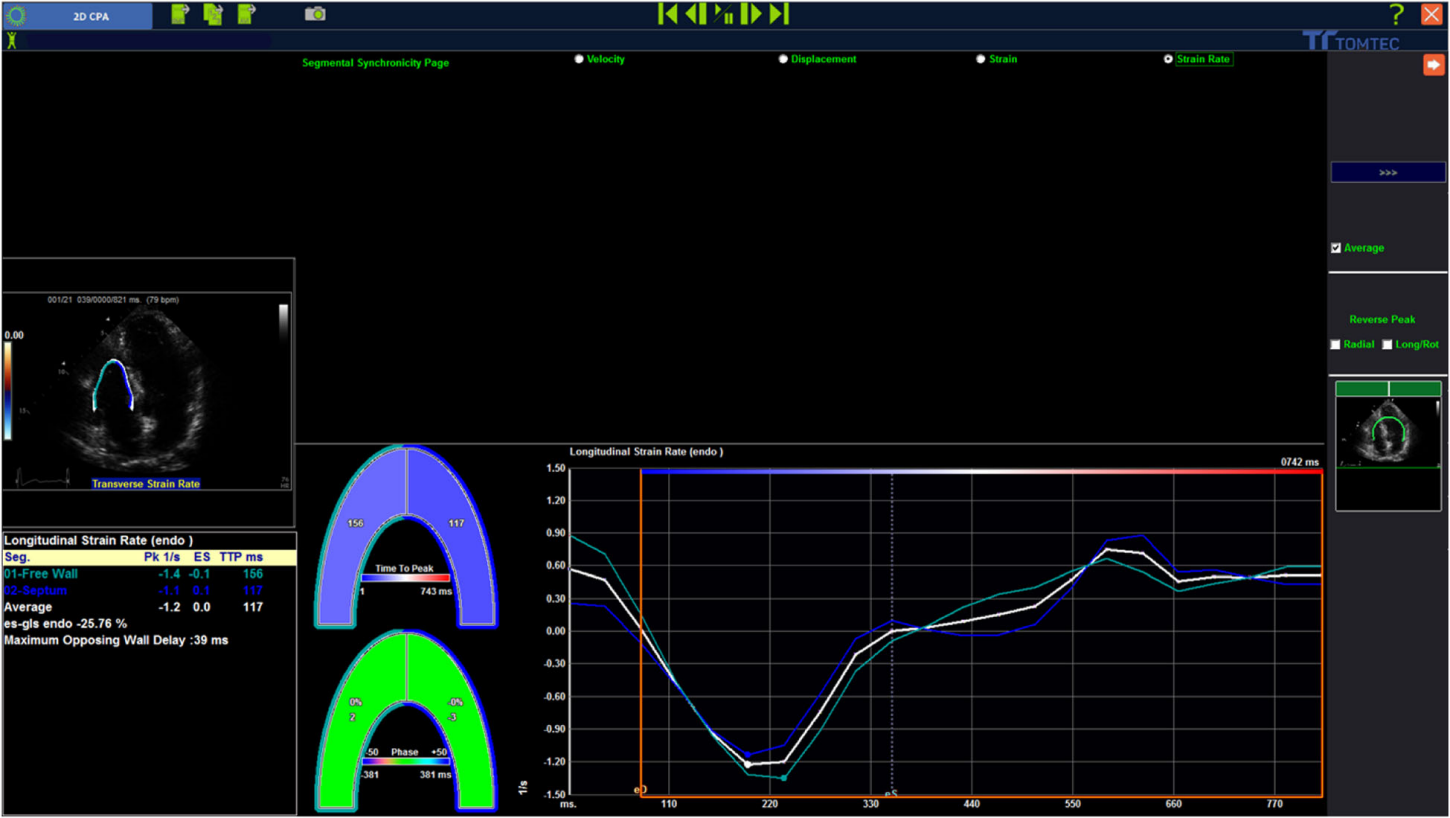
RV strain analysis using the TomTec software (2D cardiac performance analysis) for RVGLS and RVFWS by speckle tracking echocardiography. Mean frame rates = 70–90 frames/s. Digitally stored for 3 cardiac cycles. Endocardial border tracking was achieved automatically using 2 points in the annular region and 1 point in the apical segments. Tracking quality was visually verified. Segments that failed initial tracking were manually adjusted. Segments that could not be tracked properly after manual adjustment were rejected. RV GLS strain was measured from the average value of the longitudinal peak systolic strain of the RV free wall and the RV septal wall in the apical 4 chamber view. LVEF: Left ventricular ejection fraction. RVFAC: Right ventricular fractional area change. RVFWS: Right ventricular free wall strain. RVGLS: Right ventricular global longitudinal strain

**Fig. 2:**
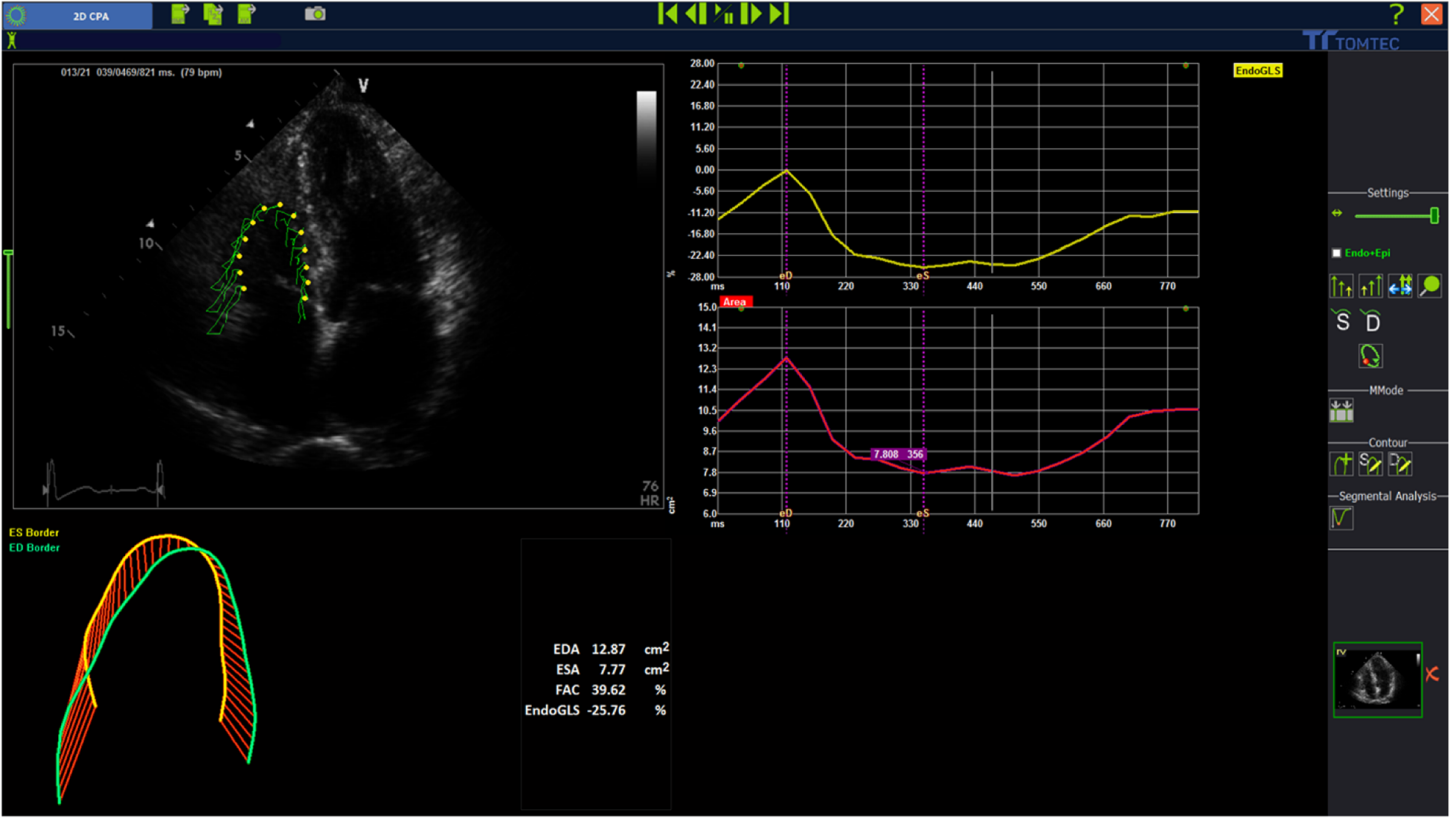
RVFAC obtained by the TomTec software (2D cardiac performance analysis) 2D speckle-tracking echocardiography. FAC was measured using RV end-diastolic area (RVDA) and end-systolic area (RVSA) obtained by manual tracing of the RV endocardium at end-diastole and end-systole in the apical 4-chamber view. The formula: [RVDA-RVSA/RVDA] × 100 was then used to calculate FAC. To limit sub-optimal interobserver reproducibility, it was ensured that the RV was contained in the whole imaging frame throughout systole and diastole, while ensuring that trabeculae in RV cavity was included. LVEF: Left ventricular ejection fraction. RVFAC: Right ventricular fractional area change. RVFWS: Right ventricular free wall strain. RVGLS: Right ventricular global longitudinal strain

STE analyses were performed according to the guidelines of the American Society of Echocardiography. The stored images were retrospectively assessed using 2D STE offline analysis software (2D Cardiac Performance Analysis) developed by TomTec Imaging Systems, GmbH (Munich, Germany) stored in DICOM format. All echocardiographic images were acquired with mean frame rates of 70–90 frame/sec and digitally stored for 3 cardiac cycles. This method has been described in detail [7–9] and involves tracking speckles from frame- to- frame. RV strain parameters: (RV global longitudinal strain [GLS], RV free wall strain [FWS]) and RV fractional area change [FAC] (Figs. 1 and 2) were determined by selecting the most representative of the 3 cardiac cycles and marking the endocardial region of interest in the standard RV-focused apical 4 chamber view. Endocardial border tracking was achieved automatically using 2 points in the annular region and 1 point in the apical segments. Tracking quality was visually verified. Segments that failed initial tracking were manually adjusted. Segments that could not be tracked properly after manual adjustment were rejected.

RV GLS strain was measured from the average value of the longitudinal peak systolic strain of the RV free wall and the RV septal wall in the apical 4 chamber view (Fig. 1).

FAC was measured using RV end-diastolic area (RVDA) and end-systolic area (RVSA) obtained by manual tracing of the RV endocardium at end-diastole and end-systole in the apical 4-chamber view. The formula: [RVDA-RVSA/RVDA] × 100 [10] was then used to calculate FAC. To limit sub-optimal interobserver reproducibility, it was ensured that the RV was contained in the whole imaging frame throughout systole and diastole, while ensuring that trabeculae in RV cavity was included (Fig. 2).

### Statistical methods

All calculations were performed using SPSS/PC statistical program (version 21, SPSS Inc., Chicago IL, USA). Continuous variables were reported as means ± standard deviation while categorical variables were expressed as numbers, percentages or ratios. The differences for continuous variables over time were analyzed using general linear model for repeated measures. Multiple regression analysis was performed to observe between cumulative anthracycline doses, and RV FAC, GLS, and FWS. Paired two tailed t-tests were applied for post-hoc analyses. Multivariate regression analysis was performed to adjust for covariates including age, body mass index (BMI), pulmonary hypertension, chronic obstructive pulmonary disease, dyslipidemia, diabetes, hypertension, and heart rate at time of echocardiography. We also studied the dose-dependent effect of doxorubicin on RV function by dividing the patients into 2 groups of low cumulative doxorubicin vs. higher cumulative doxorubicin doses, defined as < 200 mg m^− 2^ versus > 200 mg m^− 2^. Cutoff was chosen at 200 mg m^− 2^, as it is the lowest cumulative doxorubicin dose defined with significant LV systolic dysfunction, and thus, cardiotoxicity in the latest guideline consensus [2]. RV function parameters were analyzed by two independent observers blinded to the clinical characteristics of the study population. Reproducibility for strain was performed by intraclass correlation coefficient (ICC) analysis. For all analyses, *P*-value < 0.05 was considered statistically significant.

## Results

A total of 35 patients with a mean age of 51 ± 13 years, were included in the final analysis; 40% were women; 60% white, 14% African American, 8% Asian, and 8% Hispanic (Table 1). Mean cumulative anthracycline dose was 239 ± 104 mg m^− 2^ and mean follow up period was 6 months. For the measurement of RV GLS, RV FAC and RV FWS, the interobserver ICC was 0.87, 0.81 and 0.79 respectively, suggesting satisfactory reproducibility.

**Table 1:** Baseline characteristics at cumulative doxorubicin dose < 200 and ≥ 200 mg m^− 2^

		Cumulative Doxorubicin dose (mg m^**− 2**^)		
	All patients	< 200	≥200	***P***-value
**N (%)**	35	14 (40%)	21 (60%)	
**Age (year)**	50.6 ± 13.2	50.3 ± 13.4	50.8 ± 13.4	NS
**Female (%)**	14 (40.0%)	5 (14.2%)	9 (25.8%)	NS
**White (%)**	21 (60.0%)	11 (31.4%)	10 (28.6%)	NS
**Hyperlipidemia**	18 (51.4%)	5 (14.3%)	13 (37%)	NS
**Diabetes**	19 (54.2%)	10 (28.6%)	9 (27.5%)	NS
**Hypertension**	27 (77.14%)	11 (31.4%)	16 (45.7%)	NS
**Smoker (%)**	13 (37.1%)	5 (14.3%)	8 (22.9%)	NS
**COPD (%)**	3 (0.9%)	2 (0.6%)	1 (0.3%)	NS
**Heart rate (bpm)**	89.1 ± 14.2	92.8 ± 17.2	86.6 ± 11.5	NS
**BMI**	27.9 ± 5.6	28.1 ± 5.7	27.8 ± 5.7	NS

*BMI* Body Mass Index, *bpm* beats per minute, *COPD* chronic obstructive pulmonary disease

**Fig. 3:**
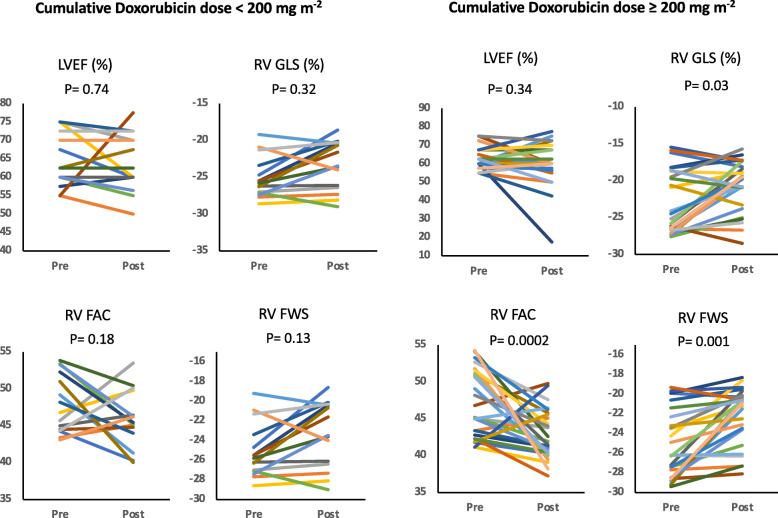
Associations between cumulative doxorubicin dose < 200 and ≥ 200 mg m^− 2^ and RV function parameters/LVEF. LVEF: Left ventricular ejection fraction. RV FAC: Right ventricular fractional area change. RV FWS: Right ventricular free wall strain. RV GLS: Right ventricular global longitudinal strain

During the mean follow-up period from pre- to post-doxorubicin therapy, there was significant decline in mean RV GLS, RV FAC and RV FWS (all p = < 0.01; Table 2). No significant decline in LVEF was observed (*p* = 0.37). We also observed a dose-dependent decline in RV function at higher (≥ 200 mg m^− 2^) vs. lower (< 200 mg m^− 2^) doses of doxorubicin from pre- to post-doxorubicin therapy; RV GLS *p* = 0.03, RV FAC and FWS *p* < 0.01 (Table 3 and Fig. 3).

**Table 2:** Changes in mean RV GLS, RV FAC and RV FWS at baseline to follow-up period from pre- to post-doxorubicin therapy at 6-month follow-up

***N*** = 35	Pre	Post	***P***-value
**LVEF (%)**	63.3 ± 6.2	61.6 ± 11.1	0.37
**RV GLS**	−22.4 ± 1.4	−20.6 ± 3.4	0.01
**RV FAC (%)**	47.3 ± 4.4	43.7 ± 3.9	0.01
**RV FWS**	−24.9 ± 3.3	−22.2 ± 2.9	0.01

*LVEF* Left ventricular ejection fraction, *RVFAC* Right ventricular fractional area change, *RVFWS* Right ventricular free wall strain, *RVGLS* Right ventricular global longitudinal strain

**Table 3:** Echocardiographic parameters pre- and post-doxorubicin therapy for cumulative doxorubicin dose < 200 and ≥ 200 mg m^− 2^

	Cumulative Doxorubicin dose (< 200 mg m^**− 2**^)			Cumulative Doxorubicin dose (≥200 mg m^**− 2**^)		
	Pre	Post	***P***-value	Pre	Post	***P***-value
**LVEF (%)**	63.8 ± 6.9	62.9 ± 7.5	0.74	63.0 ± 5.9	60.7 ± 13.1	0.34
**RV GLS**	−22.6 ± 3.6	−21.3 ± 3.9	0.32	−22.3 ± 4.5	− 20.1 ± 2.9	0.03
**RV FAC**	47.9 ± 4.1	46.1 ± 3.9	0.18	47.0 ± 4.7	42.2 ± 3.1	0.01
**RV FWS**	−25.2 ± 2.8	−23.6 ± 3.4	0.13	−24.6 ± 3.6	−21.5 ± 2.4	0.01

*LVEF* Left ventricular ejection fraction, *RVFAC* Right ventricular fractional area change, *RVFWS* Right ventricular free wall strain, *RVGLS* Right ventricular global longitudinal strain

**Fig. 4:**
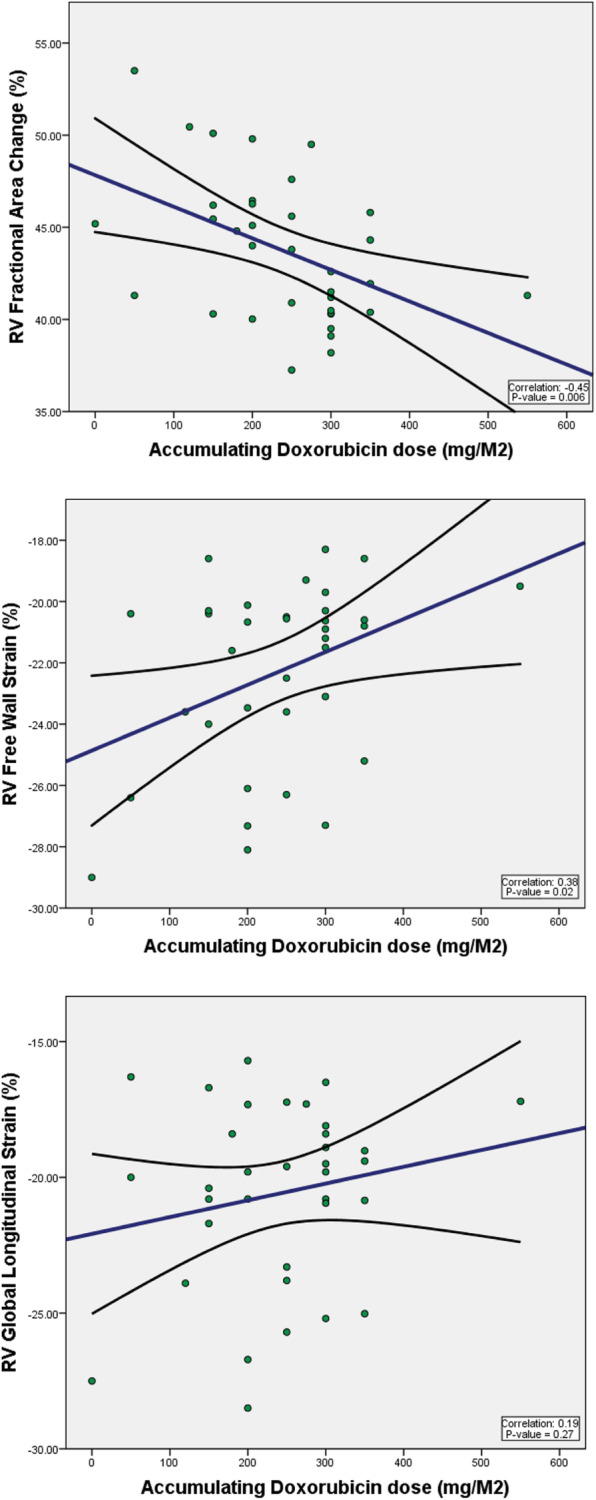
Multi-Regression Analysis for Correlation between Cumulative Dose of Doxorubicin and RV FAC, RV FWS, and RV GLS**.** Model adjusted for: age, BMI, pulmonary hypertension, chronic obstructive pulmonary disease, dyslipidemia, diabetes, hypertension, and heart rate at time of echocardiography. RV FAC: Right ventricular fractional area change. RV FWS: Right ventricular free wall strain. RV GLS: Right ventricular global longitudinal strain

Furthermore, cumulative dose of doxorubicin was significantly associated with percent decline in RV FWS (*P* = 0.028) and RV FAC (*P* = 0.045) on multi regression analysis (Table 4, Fig. 4). There was a non-significant correlation between cumulative doxorubicin dose and percent decline in RV GLS (p = NS; Fig. 4).

**Table 4:** Multi-Regression analysis of associations between cumulative doxorubicin dose and percent decline in RV function parameters

	Coefficient	***p***-value
**ΔRV FAC (%)**	0.404	0.045
**ΔRV FWS (%)**	0.420	0.028
**ΔRV GLS (%)**	0.907	0.373
Model adjusted for age, BMI, COPD, pulmonary hypertension, DLD, HTN, diabetes, heart rate during echocardiography		

*BMI* Body Mass Index, *COPD* chronic obstructive pulmonary disease, *DLD* Dyslipidemia, *Echo* Echocardiogram, *LVEF* Left ventricular ejection fraction, *RVFAC* Right ventricular fractional area change, *RVFWS* Right ventricular free wall strain, *RVGLS* Right ventricular global longitudinal strain

## Discussion

Doxorubicin-induced LV cardiotoxicity has been well-studied and established. Our study showed that in a dose-dependent manner, doxorubicin was associated with subclinical RV dysfunction measured by 2D STE, but not changes in LVEF, in lymphoma patients with no known baseline heart disease. To our knowledge, our study is one of the first of its kind to show that subclinical RV dysfunction parameters were associated with higher cumulative doses of doxorubicin even though LVEF was not. Zhao et al. recently demonstrated subclinical changes in RV function parameters after 6 cycles of anthracycline chemotherapy [11]. Furthermore, our study is the first to assess RV FAC as a significant measure of RV function in this setting. RV FAC has been shown to be an independent predictor of heart failure [12] and an independent predictor of RV EF using cardiac magnetic resonance (CMR), and by reflecting both longitudinal and radial components of RV contraction [7].

Early detection of cardiotoxicity and prevention of cardiovascular events is especially challenging in cancer patients receiving cardiotoxic chemotherapy. Several studies have demonstrated the importance of limiting anthracycline dose to minimize cardiotoxicity and cardiac mortality [13]. Since each individual responds differently to cumulative anthracycline dose, empiric dose limitation/modification could lead to premature discontinuation of an effective anthracycline therapy [14]. Furthermore, early detection of cardiotoxicity remains the most commonly used method to reduce cardiac morbidity and mortality and novel sophisticated methods have been investigated for this purpose [15]. Indium-111-antimyosin scintigraphy and Iodine-123 meta-iodobenzylguanidine MIBG scan are demonstrated sensitive tools for early detection of anthracycline cardiotoxicity; however, they are not readily available in most clinical centers. Thus, echocardiography and specific cardiac biomarkers remain the most widely used clinical methods to detect cardiotoxicity.

The conventional criteria for diagnosis of chemotherapy-related cardiac dysfunction relies on changes in LVEF, which has high measurement variability and is not a prognostic indicator [16]. However, observational data have demonstrated LV GLS to be a sensitive marker of cardiac dysfunction. Some studies have reported that LV GLS combined with cTnT provides a reliable and non-invasive method to predict cardiac dysfunction in patients receiving anthracycline-based chemotherapy [3]. Others showed that a decrease of 10% in the peak longitudinal LV systolic strain at 3 months after chemotherapy could predict cardiotoxicity 3 months later [16]. Furthermore, LV GLS has been shown to be an early predictor of later decline in LVEF [17].

The RV plays an increasingly important role in cardiac-related morbidity and mortality in patients with cardiopulmonary disease, and RV dysfunction is associated with worse outcomes [18]. However, the assessment of the RV has been problematic and largely qualitative because of difficulty with assessing volumes due to its unusual shape. In our study, we used 2D STE to overcome these limitations. 2D STE of the RV is a relatively new technique not influenced by angle-dependency and tethering effect, as its improved signal-to-noise ratio overcomes most Doppler limits [19]**.** Though less established than FAC, the advantage of RV GLS and FWS measurement is that the measurements are both angle-independent and less sensitive to loading conditions [20]. Due to these properties, RV GLS and RV FWS correlate more robustly with RV systolic dysfunction than the conventional parameters of TAPSE, tricuspid s’ velocity, and RIMP [7, 20].

In our study, decline in RV FWS and FAC but not RV GLS was strongly associated with cumulative doxorubicin dose. Although non-significant, there a trend in correlation showing more abnormal RV GLS with higher doxorubicin dose (Fig. 4). In comparison to RV GLS, RV FWS correlates better with RV EF by CMR and has greater prognostic value in multiple disease states, such as heart failure and pulmonary hypertension [21]. Thus, recent recommendations from ASE call to standardize deformation imaging by using RV FWS as the default measure of strain [20].

Cumulative anthracycline exposure has been studied and is largely associated with the risk of cardiac toxicity [14, 22]. We found for the first time, a dose-dependent subclinical RV dysfunction associated with doxorubicin. According to guidelines, a baseline echocardiographic study should be performed before the administration of anthracyclines. The next assessment should be performed at 250–300 mg m^− 2^, then at 450 mg m^− 2^ [23]. Our study found subclinical RV dysfunction even without a decrease in LVEF 6 months after doxorubicin therapy and suggests that subclinical RV dysfunction could serve as an early warning sign for chemotherapy planning. RV strain is increasingly found to be an independent predictor of outcomes, as well as having stronger prognostic power than standard LV measurements in heart failure [10].

Our study was limited by its small sample size with limited power, which implies that our findings, including strain and dose-dependent trend towards increased all-cause mortality, could be even more robust with a larger sample of patients. The retrospective nature of the study likely adds some bias to our study findings and limits clinical correlations due to non-standardized clinical assessments that lack longitudinal correlation to the strain parameters assessed. We also could not assess associations between RV strain parameters and prognostic biomarkers such as high-sensitive troponin or natriuretic peptides. As this is primarily an imaging study, subclinical cardiac status was the focus of correlation.

## Conclusions

In this cohort of adult lymphoma patients without known heart disease, despite absence of change in LVEF, we demonstrated the dose-dependent effect of doxorubicin therapy on RV subclinical dysfunction by 2D STE. 2D STE is a sensitive and reliable non-invasive method that can detect subclinical LV dysfunction in cancer patients receiving cardiotoxic chemotherapy, before a reduction in LVEF. RV dysfunction is an independent prognostic marker and a predictor of mortality in patients with cardiomyopathy and heart failure. Based on our study findings, we propose that in addition to LV, RV function monitoring may be a cost-effective tool for early detection of subclinical cardiac dysfunction during chemotherapy. Additional studies evaluating the long-term prognostic implications of RV dysfunction in this population are essential. In the future, larger prospective studies that focus on the evaluation of RV strain parameters in correlation with both biomarkers and LV strain are paramount to the validation of this study in the setting of doxorubicin chemotherapy.0.

## Data Availability

The datasets used and analyzed during the current study are available from the corresponding author on reasonable request.

## Abbreviations

BMI: Body Mass Index
COPD: Chronic obstructive pulmonary disease
DLD: Dyslipidemia
Echo: Echocardiogram
LVEF: Left ventricular ejection fraction
RVFAC: Right ventricular fractional area change
RVFWS: Right ventricular free wall strain
RVGLS: Right ventricular global longitudinal strain
